# Respect, Relationships, and “Just Spending Time with Them”: Critical Elements for Engaging Aboriginal Students in Primary School Education

**DOI:** 10.3390/ijerph19010088

**Published:** 2021-12-22

**Authors:** Rosalie D. Thackrah, Dawn Bessarab, Lenny Papertalk, Samantha Bentink, Sandra C. Thompson

**Affiliations:** 1 Western Australian Centre for Rural Health, University of Western Australia, Crawley 6009, Australia; lenny.papertalk@uwa.edu.au (L.P.); samantha.bentink@uwa.edu.au (S.B.); sandra.thompson@uwa.edu.au (S.C.T.); 2 Centre for Aboriginal Medical and Dental Health, University of Western Australia, Crawley 6009, Australia; dawn.bessarab@uwa.edu.au

**Keywords:** Aboriginal education, Aboriginal/mainstream partnerships, social determinants of health, student engagement, collaborative educational interventions, culturally responsive schools

## Abstract

While disparities in educational outcomes for Aboriginal children have narrowed in early childhood education and for Year 12 completions, these positive trends are not replicated in the intervening years where attendance, reading, writing, and numeracy targets have been missed. Erratic attendance in the primary years has the greatest impact on achievement; literacy and numeracy scores decline as absences increase. Family functioning and health, caregiver expectations, past encounters with the education system and socio-economic disadvantage are all implicated in poorer rates of attendance. In response to community concerns, an Aboriginal/mainstream partnership was forged in 2011 and began work in 2016 to address patterns of attendance and achievement among Aboriginal primary students in a regional city in Western Australia. This paper describes the innovative, community-led “More Than Talk” program and presents findings from teaching and support staff interviews two years after implementation. Qualitative methods were employed to analyse the data, develop themes, and ensure rigour. Findings highlighted the cascading impact of erratic attendance and the role of strong relationships, respect, and investment of time with children as critical elements in student engagement and wellbeing. Community-led, collaborative educational programs have the potential to positively impact Aboriginal students’ engagement and contribute to culturally responsive environments. If sustained, such efforts can enable learning to flourish.

## 1. Introduction 

Australia’s annual “Closing the Gap” report charts progress made on government commitments to address disparities in health and life expectancy for Aboriginal and Torres Strait Islander peoples [1]. Since its inception in 2008, it has delivered mixed results on set targets including health, education, housing, employment, and incarceration. In 2019, a new “Partnership Agreement on Closing the Gap” was established to prioritise genuine partnerships between federal, state, and local governments and Aboriginal people represented by the Coalition of Aboriginal and Torres Strait Islander Peak Organisations. This approach, which was formalised in 2020, recognises the expertise and lived experience of Aboriginal community members, and the importance of a shared decision-making process to effect meaningful change [2]. While it is too early to determine whether this agreement will reform the recalcitrant history of missed targets, it has injected fresh optimism into a process that has consistently failed to deliver in the past [2].

Aboriginal/mainstream partnerships have been the cornerstone of numerous, successful, community-based programs aimed at improving health outcomes and are recommended to guarantee Aboriginal participation in decision making [3,4,5,6]. It has been noted, however, that the factors that strengthen or derail their success are not well understood [7]. Relational aspects of collaboration are critical to the success of these partnerships; they must be sustained and underpinned by trust and integrity [3,4,7]. Flexibility, shared and clearly articulated goals, documentation of respective roles, strong leadership, and a strengths-based approach have also been identified as key components of successful partnerships arrangements [3]. Challenges that can impact negatively on partnerships include the legacy of colonial history and associated distrust, a disinclination towards power sharing with Aboriginal people, short funding cycles, and high turnover of personnel [3,4,7]. 

With awareness of these factors in mind, and intent upon building a strong relationship with the local Aboriginal community, a partnership was forged between the Midwest Aboriginal Organisation Alliance (MAOA) and the Western Australian Centre for Rural Health (WACRH), University of Western Australia, both located in Geraldton, 420 kilometres north of Perth. The collaborative health promotion project entitled, “More Than Talk: An Aboriginal, non-Aboriginal partnership for action” aimed to support the delivery of better health and wellbeing for Aboriginal people residing in Western Australia’s Midwest region and identify and strengthen Aboriginal/mainstream partnerships [8]. MAOA’s overarching objective is to encourage wider community engagement with local Aboriginal issues by working collaboratively with government, industry, and community partners while WACRH seeks to improve rural, remote, and Aboriginal health through education, research, and community partnerships. 

The “More Than Talk” health promotion project was funded through a Western Australian “Healthway” research grant and implemented between 2012 and 2017. The areas of *housing* and *education* were identified by MAOA as their priorities for research; important social determinants of health which require greater attention, both in terms of research to better understand the impediments to achieving better outcomes, and action to implement community-led initiatives [8]. While the early years of the project focused on the research dimension, the translation of key learnings, particularly around Aboriginal/mainstream partnership approaches to policy and practice were prioritised in the later years. This paper explores one of the projects’ later educational initiatives: a multi-pronged, collaborative, community-led program implemented in a local high-needs primary school in 2016–17.

Efforts to address disparities in educational outcomes for Aboriginal children have focused on participation in early childhood education, regular school attendance, narrowing the gap in reading, writing, and numeracy and increasing the rates of Year 12 completion [1]. Regular participation in early childhood education can mitigate developmental vulnerability arising from a disadvantaged home environment and is associated with improved performance at school [1]. In 2018, 86% of Indigenous four-year-olds were enrolled in early childhood education programs; this exceeded the projected target, and together with relatively high and stable attendance rates, is a very positive outcome. At the other end of the educational spectrum, Year 12 attainment rates have increased from 45% in 2008 to 66% in 2018–2019, making students more competitive in the employment market and better able to work in high-skilled occupations [1]. These positive trends, however, are not observed in the years between early childhood education and Year 12, where attendance and reading, writing, and numeracy targets have been missed [1].

While many Aboriginal children attend school on a regular basis, across Australia overall attendance rates remain 10% lower than for non-Indigenous children (82% versus 92%) and have not improved over the last five years, despite a target to halve the gap [1]. Reasons for erratic attendance are not consistently reported, however, family functioning and health, caregiver expectations, past encounters with the education system, and socio-economic disadvantage have been implicated in these patterns [1,9,10,11,12]. It has been noted that for many Aboriginal Australians, formal education was used as a tool of colonialism, initially through exclusion from classrooms and later due to assimilation policies [9,10,11]. Stories transmitted across generations have contributed to alienation and mistrust of an education system, which until very recently did little to assuage Aboriginal families’ concerns [9,10]. Drawing on interviews with Indigenous and non-Indigenous educators in a regional town in the Kimberley, Western Australia, Prout Quicke and Biddle found that feelings of inadequacy and isolation experienced in alienating school environments were further compounded by home life challenges; this likely increases disengagement and contributes to poorer patterns of attendance among some Aboriginal children [9]. Negative school experiences have been endured by many First Nations’ populations, including those in North America and New Zealand with a similar settler colonial history [9].

It is widely recognised that poor attendance during the primary years has the greatest impact on achievement; literacy and numeracy scores decline as absences from school increase and this impact is exacerbated by remoteness [1,12]. Aboriginal children’s attendance patterns tend to decline in the later years of primary school and this pattern continues and often intensifies in the secondary years, where missing foundational content can have a severe impact on educational achievement [1]. As with attendance, the national target to halve the gap for Aboriginal children in reading, writing, and numeracy by 2018 was not met. Despite some improvements, 20% of students in Year 3 and 25% of those in Year 5 are below national minimum standards in reading, with similarly poor outcomes in numeracy [1].

These findings suggest that despite concerted efforts that include targeted funding, fresh approaches are required to address patterns of attendance and achievement among Aboriginal children, and these will require community support, participation, and leadership. In a review of literature exploring Indigenous cultural awareness in the teaching space Krakouer noted that culturally responsive teaching practices have been shown to improve educational outcomes [11]. However, awareness of the heterogeneity of Indigenous groups must always be considered when frameworks are designed and working with local groups is recommended [11]. The need for fresh approaches is also acknowledged in the new Partnership Agreement between the Government and the Coalition of Peaks; it has also been recognised for some time by those working collaboratively with Aboriginal communities to effect change [3,4]. The “More Than Talk” project was one such initiative that was developed in response to community concerns around Aboriginal children’s educational outcomes and designed as an Aboriginal /mainstream partnership. Two schools with high needs were identified as likely to benefit from additional support and resources; one was remotely located and the other served a disadvantaged area of a regional city in Western Australia and is the focus of this paper.

### “More Than Talk” Program: Aims, Activities, and Personnel

In collaboration with the school leadership team, the “More Than Talk” educational program aimed to provide extra support and resources to Aboriginal children, some of whom were not regular attenders. Disadvantaged children’s home and community environments can result in the prioritisation of other needs, which in turn can impact regular school attendance. Social problems arising from poverty, overcrowded homes, transient family members, drug and alcohol misuse, and family violence are stressors that children bring to the classroom and can also overwhelm and prevent the engagement of families with the education system [13]. Aboriginal parental engagement is affected by many factors, including the impact of past policies and practices where schools were often not welcoming and expectations for achievement among Aboriginal children were low [14]. The parental and volunteer support which occurs in advantaged areas is less likely to be present in high-needs schools, where the need for extra assistance is often greater. The “More Than Talk” program attempted to address these issues by directing additional support and resources to areas where children were likely to benefit. The scope was wide and included creative activities, behaviour modification and resilience strategies, social-emotional referrals and home visits, assessments conducted by supervised occupational therapy students, a school breakfast program, and observance of cultural days of significance within the school community.

An Aboriginal project/research officer was appointed to help steer the introduction of the initiatives into the school and provide cultural advice as required; the appointee was well known in the community and had strong links with the school which spanned over 15 years. In addition, the program utilised the services of an experienced social worker with mental health training who was working for WACRH; she provided social and emotional support to children, liaised with families and health services, and offered advice to teaching staff on strategies to support children in need. A performance artist who ran creative activities with students, including breakdancing and mural painting, was also part of the team. The school was also a site for occupational therapy clinical placements where supervised students provided services which are difficult for families to source locally. The placement students practised clinical skills and learned cultural competencies while simultaneously supporting teaching staff with child assessments, recommendations, and referrals. 

Lastly, the school breakfast program, which had operated for many years was given additional support by program team members who saw it as an opportunity to do more than provide a nourishing breakfast and educate about healthy food options. They ensured that the students who attended were welcomed to the school and modelled caring about others, social engagement, and emotional support. All activities were supported by the school leadership team, teaching and support staff, the local Aboriginal and Islander Education Officer (AIEO), and the Aboriginal Education Regional Consultant. Most activities were designed and implemented by the program team members in collaboration with MAOA and WACRH and were favourably regarded by the local community. 

This paper focuses on the ‘More than Talk’ program offered to a regional school where Aboriginal children comprised approximately 50–60% of the school population [15]. It is informed by literature suggesting that elements of school culture and leadership that support Aboriginal children and their families can lift rates of attendance and improve learning outcomes. These elements include awareness of families’ social circumstances, a willingness to engage with and invite community participation in the provision of education, and expansion of intensive learning support where required [13,16,17]. The paper identifies the aims, scope, and collaborative nature of the “More Than Talk” program and the specific activities undertaken at the school. It also presents findings from staff interviews, which explored barriers to regular attendance and educational attainment, and the program’s strengths and limitations. 

## 2. Methods

In late 2017, 2 years after its implementation, interviews were held with teaching and support staff of the school involved in the program, the school leadership team (principal and two deputy principals), and Aboriginal departmental officers to determine the effectiveness of the intervention strategies. Nine in-depth interviews were conducted with eleven participants; eight were held individually and a group interview was held with the leadership team. A semi-structured interview schedule was used by the interviewer. Topics covered included staff roles; challenges encountered to improve academic achievement, attendance, and parental engagement; strategies in place, successes, and areas for improvement; and feedback on the “More Than Talk” program, how it worked, and where it could be strengthened. 

Participants were identified by the principal and included classroom teachers, support staff, Aboriginal consultants, and the leadership team. Selection was purposive; it included only those who had knowledge of and involvement in the program, and all those approached agreed to be interviewed. All interviews were conducted on site and participants were given information and consent forms prior to commencement. Interviews were recorded with participants’ signed approval and lasted between 30 and 60 minutes. Transcripts were prepared by a private transcription service and subsequently kept in a password-protected computer and network drive. 

Standard qualitative techniques were employed to analyse the transcripts [18]. This involved a careful, systematic process of immersion in the data through multiple readings, detailed coding, and identification of emerging themes [18]. Initial codes were recorded and links between codes (axial coding) were established. Emerging themes arose from a deductive process that involved code categorisation, the combination of categories and attention to repeated patterns of meaning. Due to the small number of transcripts (9) qualitative analysis was conducted without the use of software. This labour-intensive process of analysis strengthened the trustworthiness and credibility of the findings and was enhanced by regular communication within the research team [18,19]. Approval for the study was granted by the Western Australian Aboriginal Health Ethics Committee (ID 671) with reciprocal approval by the Human Research Ethics Office, University of Western Australia, and the approval of the Department of Education. 

## 3. Results

The participants comprised eight females and three males who had been at the school for between 18 months and 28 years. Their roles varied from middle to upper primary classroom teachers (three), principal and deputies (three), an Aboriginal education regional consultant, an Aboriginal education officer, a chaplain, a school psychologist, and a learning support coordinator. All five support roles were part-time, the remainder were full-time.

### 3.1. Barriers to Academic Achievement among Aboriginal Children

Erratic attendance and transiency were identified by all participants as key factors that inhibit academic achievement. Other factors cited included peer relationships, behavioural issues, low literacy skills, stress, unstable home environments, limited parental engagement and parents’ literacy skills. Poor attendance and transiency impacted confidence, friendships, and relationships with teachers, all essential for learning to occur.

*Because they haven’t engaged at school, they don’t have friendships, they don’t know what’s going on. They have got big gaps in their learning, so when they get here… they don’t feel safe, they feel uncomfortable, they feel distressed. They don’t know the routines, don’t have a relationship with the teacher, and can be a bit more aggressive with social interactions.*

It was recognised that children’s attendance at school reflected their family’s circumstances. Staff noted that transiency often occurs when families move temporarily to live with others if support is needed, when “sorry business” arises, or as a response to housing insecurity; children can miss school for many weeks, only returning when circumstances change. For less transient families, other factors were seen as affecting children’s regular attendance. These included parents’ past experiences with the education system, the relatively young age of parents, and the myriad of personal and social problems that accompany socio-economic disadvantage; in these circumstances education is not always prioritised.

Erratic attendance and associated academic struggles were seen as intricately linked to behavioural problems and peer relationships. Those who were not coping often displayed high levels of stress and anxiety, which in turn affected behaviour in the classroom and playground. While aggressive behaviour was observed occasionally, more likely was that children retreated into themselves and became isolated. It was suggested that these children mostly seek acceptance and reassurance; they need coping strategies and adults to spend more time with them. It was noted that when at school some craved and sought one-on-one attention.

Limited parental engagement was viewed, in part, as an outcome of low levels of trust with past negative experiences of schooling by parents and grandparents likely shaping future interactions. It was widely reported that many Aboriginal caregivers are reluctant to enter the school grounds and engage with teachers, thus removing informal opportunities to discuss students’ progress and attendance if required. 

### 3.2. Improving Academic Achievement among Aboriginal Children

A warm and welcoming environment where children feel safe was recognised as a precondition for academic achievement and encourages regular attendance. One teacher noted that:

*…the first and foremost thing is respect and relationships… not kind of assuming that because you’re the teacher, you’re very important. It means talking to the kids when it’s not duty time and actually being interested in what they’re doing… asking them about their lives. That relationship-building happens a lot at this school.*

While patience was considered essential to relationship-building, clearly defined boundaries, which create a safe environment and promote independence were also seen as necessary. Several participants noted that teachers’ awareness of the social world children inhabit is important and encouraged. *“Kids appreciate that we know who they are, their families, the histories… they like those connections, like us knowing older siblings”*. An ability to make learning fun was also seen as an important component of successful learning. Staff described numerous initiatives to address academic achievement while also recognising that solutions are often outside their control. Intervention groups for literacy and maths, attendance awards, involvement of Aboriginal and Islander Education Officers (AIEO), Individual Education Plans, a Positive Behaviour Schools program, and early work on an Aboriginal Cultural Standards Framework (ACSF) that incorporates cultural content into the curriculum, were cited as attempts to bolster academic achievement. An Aboriginal staff member noted that the ACSF should be guided by *“an educator with cultural authority”*. There was general agreement that local cultural leaders need to be closely involved in the process and that teachers need opportunities to become better informed about the history, culture, and language of local Aboriginal communities. 

While it was acknowledged that parental engagement with a school can positively influence students’ patterns of attendance, sense of security, and academic achievement, participants acknowledged numerous barriers arising from distrust and described efforts made by the school to be more inclusive and welcoming of parents. These included welcome barbeques with chocolate wheels and raffles, parent evenings, a ‘learning journey’ initiative where families visit classrooms without formal parent interviews and invitations to sporting events, which were the most well attended of all the initiatives. There remained reluctance to engage however, and some participants contrasted parental engagement with the school to that at a local Aboriginal community centre where parents are more likely to be keen participants.

A number of suggestions were offered about what more the school could do to improve Aboriginal children’s academic achievement and parental engagement. Building community relationships and working with families and Elders were identified as areas that need to be guided by Aboriginal community members and required work. It was also noted that the sharing of positive experiences and successes are important; there can be too much emphasis on communicating problems. Informal connections such as: *“putting my head through nan’s car window and talking to her about the kids, it was all positive”*, often have the greatest impact. It was noted by members of the leadership team that to improve the relationship with parents the school had adopted a strengths-based approach which included a commitment to send out letters of commendation and conduct positive home visits. Several participants also noted the need for more intensive literacy and numeracy programs. 

### 3.3. “More than Talk”: What Worked Well?

Participants valued the regular presence of three additional skilled adults and the steady stream of occupational therapy and other students and supervisors which added considerably to the resources available within the school to support needy children. These WACRH staff were recognised as part of the school but staff respected their creative autonomy, which gave them freedom to explore new ways to connect with children and their families. It was recognised that they had developed links with the local Aboriginal community and by building trust were better able to break down communication barriers.

It was suggested that the inclusion of a social worker on the team facilitated difficult home visits and the collection of more background information on children. The social worker was viewed as a powerful presence in classrooms where cognitive behaviour and resilience programs were conducted; key messages were also reiterated in the playground. One teacher noted that: *“my attendance is best on that day. With xxx it’s personal and therapeutic… the kids need strategies to cope with the problems, but they also need those people that are just going to spend time with them”.* It was recognised that the social worker quickly established trust with the children and that they were constantly *“seeking her out”.* Issues were identified and acted upon quickly, including socio-emotional referrals, which freed up time for other support staff to focus on academic performance. The work undertaken and the value attached to it were seen by the leadership team as evidence to support the argument for an on-going social worker position in high-needs schools. 

Another highly successful component of the program identified by staff was the work undertaken by the performance artist who provided a positive role model for the boys and injected fun and creativity into activities with the children. *“He was engaging the kids in the arts but also helping them have a sense of ownership with the school… two kids in particular, I’ve noticed a big change in attitudes and behaviour”.* It was noted that both the social worker and performance artist had a certain magnetism with the children who, in turn, sought their attention. The following success story was described by one of the participants:

*What I’ve noticed with (this school) is that it’s always extremely welcoming, and I think having xxx and xxx there, well it’s made a huge difference to some of those kids. A classic example was last year before More Than Talk really came on board, the office at lunchtime or morning recess used to be filled with kids that had done stupid things… now you are lucky if you get three or four a week. The kids know they’ve got a safe place, they can go and talk to someone other than their teacher… and it’s hard for teachers with so many kids.*

*The disabled boy with a prosthesis, he was always in trouble, but really connected with xxx, he dances and carries his things for him. And the little year one girl, all she did was cry and cry and cry, and then xxx started talking with her… and over time, well, she now walks around with a big smile on her face and mixes with the other kids. I think that all those sorts of things are accolades for your program.*

The additional resources provided by the occupational therapy students on clinical placements were also identified as a valuable aspect of the program as they could offer small group and one-on-one assessments and assist with the referral process. It was noted that initiatives including the expanded breakfast program and the NAIDOC (National Aborigines and Islanders Day Observance Committee) Day celebrations in the school galvanised community support. The Aboriginal project officer’s role was seen as pivotal to the success of the various activities; it was advisory, consultative, and grounded in knowledge of the barriers to engagement and awareness of community aspirations as well as detailed knowledge of the children and their family circumstances. The celebrations in particular were referred to in glowing terms by numerous participants who recognised the importance of embedding Aboriginal cultural knowledge and significance events into the daily rhythms of the school and the role they play in encouraging Aboriginal families to become involved. 

### 3.4. “More than Talk”: Areas for Improvement

While the program and associated collaboration with WACRH was highly valued, a few areas were identified for fine-tuning. First and foremost was the need for a heightened awareness of protocols surrounding interactions with primary school students. School staff are cognisant and alert to any potential blurring of boundaries such as becoming too friendly with children. In this context the need for *“crossing t’s and dotting i’s before you get involved”* was raised. It was suggested that students need more orientation to heighten their awareness of appropriate boundaries.

Several participants raised concerns about children’s potential dependence on one-on-one interactions, which were sometimes available with program staff and students. One classroom teacher noted, “*the kids are craving it (the attention), you can fill them up, but you don’t want to create a situation where they’re dependent to the point where they can’t function without that support. It needs to be set up and managed in a way to promote independence, so they learn to rely on themselves.*” It was suggested that if children become too reliant on individual attention, they can snap when classroom teachers failed to provide it. This was seen as a potential risk needing management. 

It was suggested that another limitation of the program related to its short duration, which makes evaluation more difficult. While some evidence was provided of improvements in children’s attitudes, behaviour, and attendance during the duration of the program, the timeframe was too short to establish a conclusive link and disentangle the impact of other school-based initiatives. Longer-term implementation was considered more desirable to better gauge the success of the program and to avoid the *“churning”* issue where *“kids like it but know it’s short term”.* However, it was understood that programs are dependent on funding and their sustainability is not guaranteed. Finally, some participants drew attention to “in-house” improvements and encouraged wider dissemination of program activities as not all staff were aware of the range of activities available. 

## 4. Discussion

The “More Than Talk” program was a collaborative, community-led response to local concerns about Aboriginal children’s educational outcomes. It was implemented in a high-needs, regional primary school attended by many children from disadvantaged backgrounds where, for a range of reasons, education achievement may not be prioritised. Improvements in Aboriginal children’s educational outcomes have been observed where schools provide supportive environments for learning, offer social and emotional support to children in need, and engage with the local community to ensure that Aboriginal culture is respected and incorporated into the school ethos [13,20,21]. While “out of school” factors such as unstable home environments can negatively impact achievement, school-based initiatives that are collaborative and community-led provide opportunities to ameliorate some of the effects of social disadvantage [13,17]. 

Three main themes arose from interviews with participants involved in the “More Than Talk” program. These include: the cascading effects of erratic attendance, improving engagement through community-led interventions, and celebrating successes and building on achievements.

### 4.1. The Cascading Effects of Erratic Attendance

While most Aboriginal students are regular attenders, the erratic attendance of some was described as a “downward spiral” that affects confidence, peer and teacher relationships, behaviour, and academic achievement. Transiency, where families move for extended periods of time before returning has a similar effect upon children, as schooling is disrupted. Despite efforts by teachers to support those who miss classes, their time is limited. The cumulative effect of constant absences can result in low levels of achievement, disinterest in schoolwork, and social isolation; it also creates additional workload and is stressful for teachers who are already time poor. Regular attendance enables students to maintain connections with peers and teachers and keep pace academically [17].

Dreise [22] (p.1) and colleagues noted that despite a range of levers that can be applied to address poor school attendance, including investment in community programs, “public policy and political discourse… has, in recent years, been framed within the context of behavioural change by parents of Indigenous young people, namely, through welfare reform.” They suggest however, that “top-down” approaches are “unlikely to have sufficient impact by themselves, especially when they are devoid of ownership, leadership and action at a community level” [22] (p. 1). Such approaches can disempower and alienate communities and hence are more likely to fail. Instead, it is argued that school attendance must be considered more holistically as a broader social policy challenge not merely an educational challenge; strengthening attendance requires “highly focused policy design, targeted programs and coordinated efforts at local levels” [22] (p. 2).

Children miss school for a range of reasons; a combination of home, school, and individual factors are usually implicated [17]. Researchers often attribute poor attendance to structural factors associated with disadvantage: poverty, poor health, family stress, and geographical location, particularly in the case of secondary students [12]. While there is increasing recognition of the impact of trauma on children’s behaviour and ability to engage and learn [23], schools remain challenged by lack of diagnosis of health, behavioural issues, and learning difficulties in children, and poor access to services including paediatricians and allied health support. While remoteness is inversely related to regular school attendance, in urban and regional areas too, locational disadvantage has been observed [22]. In this study in a disadvantaged area of a large regional centre, teachers and support staff recognised that some causes of poor attendance lie outside their control; however, they were also cognisant of the school’s responsibility to provide meaningful opportunities for children to fully engage in learning and to build strong relationships with the local community. 

### 4.2. Improving Engagement through Community-Led Interventions

Winch [24], in a recent doctoral study exploring Aboriginal student engagement in three Victorian primary schools, identified increased levels of engagement as critical to improved academic outcomes. Findings revealed that transiency, racism, and a lack of connection with peers impede school engagement, while culturally safe school environments that celebrate Aboriginal culture, sport, and offer peer support, facilitate engagement and lead to better academic outcomes [24]. Efforts to increase engagement also need to consider the experiences of parents and caregivers who may harbour distrust of an education system which had failed them as children. 

With reference to attendance, Dreise, and colleagues [22] (p.17) noted that “coordinated interaction is required both inside and outside the school gates”; this also relates to student and parent/caregiver engagement where it is incumbent on schools to develop positive community relationships if attendance is to be strengthened. It has been suggested that bridge-building between public agencies and community-led organisations is a hallmark of successful educational interventions [17]. While pre-dating the new “Closing the Gap” partnership agreement which emphasises community collaboration and empowerment, the “More Than Talk” program anticipated this shift; it was developed collaboratively as an Aboriginal/mainstream partnership and incorporated most features that lead to successful partnerships, including strong leadership, flexibility, a strengths-based approach, and recognition that given the resources and support, Aboriginal community members are best placed to address their own problems [3,4,7]. 

The additional resources and activities provided by the program were valued by all participants; they recognised that team members brought specific skills to their interactions with children and families. These skills include socio-cultural knowledge that arises from immersion in community, capacity to connect with children through creative activities, and the application of social work professional skills to address children’s social and emotional needs. School staff benefited by having “more hands on deck” including students on placement, the provision of advice that is locally informed, and children who are often more engaged with learning and happier. While areas for fine-tuning were identified, it was considered that the program achievements, including an influx of Aboriginal parents during NAIDOC Day celebrations, outweighed any limitations.

### 4.3. Celebrating Successes and Building on Achievements 

Teachers and support staff involved in the “More Than Talk” program made frequent reference to the culturally responsive, welcoming, and community-focused nature of the school leadership team; indeed, without these attributes the program may not have been implemented in the first place. Parallel initiatives addressing Aboriginal children’s educational outcomes existed within the school, with some successes evident. A consistent theme among participants was that despite setbacks, the attendance and achievements of many children is sound, and improvements that occur should be celebrated more often. The whole school approach was positive and supplements those programs commonly focussed on problematic or at-risk children. It aligns with the strength-based approaches favoured for improving Aboriginal outcomes and provides a platform on which to build further achievements [25]. An example cited of this approach was the concept of “positive home visits” where students’ achievements are recognised and the usual purpose of “visits from the authorities” reversed.

Another strengths-based pedagogical approach that has been trialled among Aboriginal primary school children is the innovative “on country learning” project in Whadjuk Noongar country in south-west Western Australia. Designed collaboratively with Aboriginal community members including Elders, the project aims to imbue students with local cultural knowledge, deepen their cultural connections with land and “foster a robust sense of cultural identity and well-being” [21] (p.86). These characteristics are not only protective of emotional health but when offered as a school initiative, can also impact on school engagement, attendance, and teachers’ cultural capabilities [21]. Preliminary evidence from the program highlights the multitude of benefits that can flow from community collaboration and its potential application to other schools with high Aboriginal enrolments [21].

A recent systematic review of school-based programs that aim to build resilience and coping skills among primary students found that teacher involvement in program delivery, and teacher and program adaptability characterise successful intervention programs [26]. While the authors observed the positive impact of mental health promotion programs on students’ ability to manage daily stressors and their capacity to support all children, vulnerable children do require on-going wrap-around services with participatory, innovative, and culturally responsive approaches to realise long-term benefits.

Participants in this study were receptive to fresh approaches and keen to build upon the positive legacy of the “More Than Talk” program. Recent discussions with staff revealed interest in adopting further innovations and becoming better informed about ways to improve school experiences for Aboriginal children. Positive suggestions offered include culturally designed yarning areas in the playground, regular invitations to Elders to conduct culture and language yarning sessions with staff and students, weekly “Acknowledgement of Country” announcements, a more comprehensive approach to celebrating Aboriginal culture within the school, and the inclusion of more culturally appropriate educational resources. While still in the implementation stage, these suggestions support the school’s aims to be inclusive, welcoming, culturally responsive, and connected to the local community it serves, likely a key to students’ future patterns of attendance and achievement. 

### 4.4. Limitations

The study findings were drawn from interviews conducted with teachers, support staff, and the leadership team two years after the implementation of the “More Than Talk” educational program. Interviews with staff not directly involved in the program or from team members who participated in its delivery were not sought, as the focus of this study is upon how the program was received. 

## 5. Conclusions

The “More Than Talk” educational program was designed as part of a wider Aboriginal/mainstream partnership because educational outcomes were recognised as poorer for Aboriginal children and education was valued by Aboriginal leaders. The program was implemented in a high-needs primary school where the attendance and achievement of some students was identified as a community concern. Interviews with school staff revealed numerous strategies employed to improve student engagement. Staff were very receptive to the program; they appreciated its collaborative nature, the additional resources provided, especially program members with specialised skills and strong community ties, and the time invested in children with complex needs. While some fine-tuning was recommended, the program was highly valued, left a positive legacy and encouraged further initiatives in the school, with many aspects continuing in some form. While the short duration of the program was a limitation, further research on similar longer-term initiatives will build the evidence-base around Aboriginal-mainstream educational partnerships. Findings from this study, however, suggest that community-led, collaborative educational programs have the potential to positively impact Aboriginal students’ engagement and wellbeing, and contribute to culturally responsive environments where learning can flourish. 

## Data Availability

Data are available only on request due to privacy considerations.
